# Quality of life, mentalization, and perception of challenging patient encounters in dentistry: A cross-sectional study

**DOI:** 10.1038/s41405-023-00156-6

**Published:** 2023-07-07

**Authors:** Jan-Are K. Johnsen, Sunniva B. Haukefer, Sofie. J. Korsan, Maria Larsen, Gro Eirin Holde

**Affiliations:** 1 Department of Clinical Dentistry, Faculty of Health Sciences, UiT The Arctic University of Norway, Tromsø, Norway; 2 The Public Dental Health Service Competence Centre of Northern Norway, Tromsø, Norway

**Keywords:** Communication skills in dentistry, Occupational health, Continuing professional development in dentistry

## Abstract

**Objective:**

This study investigated how exposure to challenging patient encounters influenced participants’ self-reported quality of life, and how participants’ mentalization capabilities affected the perceptions of challenging patients encounters among Norwegian dentists and dental students.

**Materials and Methods:**

Data was collected with an online questionnaire, and a total of 165 dentists (*n* = 126) and dental students (*n* = 39) responded.

**Results:**

Participants who reported higher total exposure of challenging encounters reported lower quality of life (QoL). Mentalization tendencies affected the perception of challenging encounters with specific types of patients; critical and anxious; as well as the estimation of the total exposure to challenging patient encounters. Participants that were overconfident with regards to the mental states of others found these patient types less challenging and they reported less overall exposure to challenging patients than underconfident participants. Also, overconfident participants reported higher QoL than underconfident participants.

**Conclusions:**

Mentalization capabilities of dental practitioners interact with the perception of challenging encounters in dental practice, and how practitioners respond to these challenges. Measures should be undertaken to increase the awareness of metacognitive skills in dentistry with the dual goal of improving patient care as well as the quality of life of dental practitioners.

## Introduction

In general, healthcare settings are sometimes the scene for challenging or «difficult» interactions between health professionals and patients [1], which accentuates the importance of interpersonal skills. Central to these types of situations are the existence of some form of conflict [2] between the patients’ needs and understanding of the situation and the health professionals’ needs and interpretation of the patients’ situation. From a historic perspective it has been common practice within many healthcare professions to refer to such conflicting encounters as encounters with «difficult patients» [3]. Studies show that the “difficult patient” is also discussed frequently in relation to the dental healthcare setting [4, 5], and it appears that certain types of patients, or patient scenarios, are perceived as more stress inducing than others. For instance, when dentists were asked to rank the most intense stressors in their dental practice, anxious patients were ranked fifth among eleven possible, and fear of causing patients pain or unpleasantness was ranked second after time pressure [6]. Outside of the dental setting, patients classified as “difficult” in a medical encounter had over two times higher odds of having mental disorders [7]. Not surprisingly then, in order to handle such challenges, studies have pointed to the need for clinicians to utilize patient-centric, emphatic skill sets [8], and to engage in introspection and metacognitive activities in order to identify all contributing factors [9].

Key among the metacognitive abilities suggested by former studies is the ability to perceive and understand the mental states of patients (or others) and reflecting carefully about one’s own mental states, as they should relate closely to both motivation and overt behavior. In order to do so however the oral health practitioner needs to possess the ability to perform such tasks, which are commonly referred to as mentalization or reflective functioning [10, 11]. In sum, mentalization skills should be important to all healthcare encounters, as having “a mentalizing stance” is described as promoting a patient centered and “not-knowing” approach to patients’ mental states [12], which lends itself ideally to exploring the patients’ unique perspectives and experiences.

As could be expected, how people mentalize varies, and measuring this process is heavily reliant upon self-report, i.e., how people perceive or describe their own mentalization capabilities. While there are many theoretical approaches to understanding mentalization, research into the field of reflective functioning has suggested three distinct broader modes of mentalization: Genuine mentalization, hypermentalization, and hypomentalization. The primary distinction of these modes is the degree of certainty with which people make inferences about mental states [10, 13], and the confidence associated with these inferences. Genuine mentalization occurs when an individual acknowledges the inherent opaqueness of mental states, which implies that people have limited, but not nonexistent, insight into their own mental states as well as those of others. In terms of certainty, this state represents a midpoint between absolute certainty and uncertainty. It follows that deviations from this midpoint, in either direction, constitutes different impairments of mentalization. When individuals express extreme certainty related to mental states, this is referred to as hypermentalization. This state is characterized by individuals overinterpreting mental states [14] and feeling very confident about their own mentalization capabilities, thus resembling what is referred to as “overconfidence” in social cognition and social interaction [15, 16]. In contrast, extreme uncertainty about mental states, also referred to as hypomentalization, is associated with unclear reflection and thoughts around one’s own and others’ emotions and behaviors. For instance, individuals prone to hypomentalize may fall back on limited and simplistic explanations of behaviour, rather than take into account more complex and realistic explanatory models [13]. Thus, this represents distinct “underconfidence” with regards to social cognition and social interaction [17]. Note that the confidence term as used here does not attempt to indicate whether the mentalization tendencies lead to accurate social inferences, but rather how individuals experience being either certain or uncertain about the mental states of oneself or others. When it comes to confidence as an estimate of accuracy in social interactions, this is sometimes regarded more as a metacognitive skill that is independent of mentalization itself [18].

In the context of the current study overconfidence and underconfidence as expressed through hypermentalization and hypomentalization will be explored in the context of the encounter between patients and dental practitioners. The question then becomes what impact, if any, does these tendencies have on this specific arena of professional social interaction? The recent interest in the clinical therapeutic relationship in dental practice highlights the importance of the social interaction between patient and practitioner [19, 20]. In this light, it is key from a professional perspective to make sense of the mental states of the patient (i.e., motives, needs, emotions, etc.), as well as being able to reflect about one’s own mental state. For instance, it has been found that dentists claim to possess the ability to discern emotional reactions in their patients, for instance patients experiencing dental anxiety [6], and that claiming to identify these emotional states appeared to protect them against stress and burnout [21]. However, evidence from clinical practice questions the accuracy of dentists’ identifications of emotional states. For instance, when comparing dentists’ estimations against patients’ ratings, dentists do significantly worse when estimating anxiety and distress compared to estimations of pain [22], which suggest that complex mental or emotional states are harder to discern than acute pain that stems from specific dental procedures. Nevertheless, it is possible to hypothesize that successful utilization of mentalization abilities in the clinical encounter will have both intrapersonal and interpersonal beneficial effects, and that the opposite should be true for unsuccessful mentalization. For instance, for anxious patients beneficial effects of identifying patients’ anxiety and corresponding motivations and needs, will perhaps be most easily envisioned as stress reduction in the clinician (intrapersonal) and the professional steps taken by the dentist to address patients’ anxiety problems (interpersonal). Correspondingly, failure to successfully identify patients’ anxiety could lead to a breakdown of the therapeutic relationship. Indeed, several studies point to stressful and challenging encounters in dental healthcare as the main arena for when mentalization or similar skill sets play a role [4, 21, 23]^,^ and that the outcomes of the challenging encounters could vary based on how the health professionals choose to approach these encounters [4, 23].

Since mentalization is such a fundamental human endeavor and a key for optimal psychological functioning [16], deficiencies of mentalization have been reliably related to a host of problems and issues [13, 14]. The impact of being underconfident with regards to mentalization have been assessed in the general population, where individuals with hypomentalization tendencies have reported lower quality of life than individuals with genuine certainty levels [13]. While the reason for the association between hypomentalization and lowered quality of life should be regarded as multifactorial, it would be reasonable to assume that living with chronic uncertainty about the mental states of others will be emotionally unpleasant and stressful [24], and a potential source of social discomfort, conflict and misunderstandings. Since both overconfidence and underconfidence are equally erroneous with regards to optimal mentalization, it could be expected that similar negative consequences exist for hypermentalization. However, studies have shown the opposite for experiences of overconfidence in many realms of social life, where overconfident individuals appear to be happier [25] and enjoy advantages on the dating market [26] among other things. The bulk of evidence of detrimental effects of overconfidence predominantly appear to be related to cognitive processing and task performance, with overconfident individuals performing worse on performance oriented tasks than underconfident individuals [27, 28]. Also, there is a possibility that overconfident individuals could make unrealistic and unwarranted inferences in social situations due to being too confident about the intentions of others [29]. In a dental care setting this would constitute criticizing a patient that fail to follow preventative measures based on the idea that the patient “does not want to” follow the instructions, while failing to acknowledge that there might be many alternative reasons for the patient’s non-compliance. However, since few overconfident individuals will have insight into the underlying mentalization failure due to its implicit and automatic nature [30], and because high certainty and overconfidence in social situations ultimately feels good [25] and oftentimes are interpreted positively by the outside world [26], it can be hypothesized that living with chronic overconfidence will be less stressful in comparison to underconfidence. Finally, looking beyond mentalization failures and imbalances, the benefits of genuine mentalization would not only be minimizing the likelihood of experiencing stressful and challenging patient encounters, but also to allow for more successful coping with stress if it occurs. For instance, studies have shown that awareness of mental states is associated with resilience to stress among health care workers [31] and it has been proposed that mentalization should be regarded as a distinct coping resource when facing adverse life events [32].

In sum, there appears to be good reason to propose a link between mentalization capabilities, perceptions or experiences of challenging patient encounters, and quality of life for the dental health professional. Ultimately, the consequences of challenging patient encounters, and how these are perceived, addressed, and resolved, can have severe impact on both job-related factors, including burnout and job satisfaction, as well as constitute impairments of the life and health of dental practitioners [4, 21].

In light of this, the following hypotheses were formulated:

1. Mentalization tendencies influence dental practitioners’ perception of patients: Underconfidence about mental states is associated with increased perceived challenges in patient encounters, while overconfidence about mental states is associated with decreased perceived challenges in patient encounters.
2. Mentalization tendencies impact dental practitioners’ quality of life predictably, whereby overconfidence with regards to mental states is related to higher reported quality of life compared to underconfidence.
3. Increased exposure to challenging patients is associated with decreased perceived quality of life for dental practitioners.

## Materials and Methods

### Design and procedure

An invitation to participate in an anonymous online cross-sectional study was sent out to dentists working in public dental clinics in Norway (specifically clinics serving as practice clinics for students from UiT The Arctic University of Norway) and dental students in their 5th year of study from three Norwegian universities (the University of Bergen, the University of Oslo, and UiT The Arctic University of Norway). The invitation was distributed by e-mail by the heads of the dental clinics and administrative personnel at the participating universities, and consisted of information about the study and a link through which the questionnaire could be accessed, and only a single invitation was sent (no reminders). Data was collected anonymously using an electronic questionnaire hosted by the Nettskjema.no data collection service [33]. It is estimated that approximately 500 dentists and dental students were invited to participate in the study. Data was collected between October 2019 and January 2020.

### Measurements

The questionnaire asked about background characteristics, i.e., gender (male or female), age in years, occupation (dental student or dentist), and number of years of experience. Also, it contained two Norwegian language versions of validated psychometric scales used to measure the participants’ quality of life and capabilities for mentalization. Quality of life (QoL) was measured using the Satisfaction with Life Scale (SWLS) [34, 35]^,^ which consists of five statements regarding satisfaction with life. Participants were asked to respond to whether they agree or disagree with the statements on a Likert scale from 1 to 7. The sum score of the five statements were used to provide a measure of the participants quality of life, where high scores indicate a high degree of satisfaction with life in general. Mentalization was measured by the Reflective Functioning Questionnaire (RFQ) [10]. This scale consists of 8 statements about mental states that are scored on a Likert scale from 1 to 7 (strongly disagree to strongly agree). RFQ consists of two subscales indicating either certainty (RFQ_C) or uncertainty (RFQ_U) about mental states. High scores on RFQ_C indicate tendencies towards hypermentalizing (e.g., overconfidence regarding own and others’ feelings and mental state) and high mean scores on RFQ_U indicate tendencies towards hypomentalizing (e.g., lack of knowledge and underconfidence regarding the feelings and mental states of others). Calculation of mean scores for the RFQ_C and RFQ_U were made based on scoring instructions described in detail elsewhere [36]. Since the RFQ_C and RFQ_U scores were not normally distributed they were recoded into dichotomous variables based on median scores which provided two dichotomous variables indicating hypermentalizing versus optimal certainty levels (RFQ_C) and hypomentalizing versus optimal uncertainty levels (RFQ_U). Furthermore, in order to enable direct comparisons between hypermentalization and hypomentalization, a compound three-level variable was designed from the dichotomized variable to reflect participants’ primary mentalization tendencies. Participants were deemed to be primarily hypermentalizing, and overconfident, if they scored above median on RFQ_C (hypermentalizing) and below median on RFQ_U (normal uncertainty), while the opposite would be true for primarily hypomentalizing, and underconfident, participants ( > median RFQ_U; < median RFQ_C). Participants that scored below median on both RFQ_C and RFQ_U were designated as expressing “optimal confidence”, i.e., genuine mentalizing, and assigned as a midway point between overconfidence and underconfidence. Participants that expressed both overconfidence and underconfidence simultaneously were excluded from the analysis since this state lacks theoretical meaningfulness and might be an expression of measurement error or a lack of resolution in the compound variable.

Challenging patient encounters were measured by having the participants consider six proposed patient types inspired by former research (see Table 1 for an overview) [21]. Participants would rate how challenging they found each patient type to be on a scale from 1–3, where 3 indicated “very challenging” and 1 indicated “not challenging”. In addition, participants were asked to report the frequency of challenging encounters with different patients. This was measured from 1–4, where 1 indicated “daily encounters” and 4 “less than once a month”. Finally, participants were asked to estimate the percentage (0–100%) of their “total exposure” to challenging encounters in comparison to all clinical patient encounters.

**Table 1:** Descriptions of proposed patient types.

Patient type	Description
Critical	Patients who are critical of the therapist, or treatment, and express this by body language or speech. E.g.: Patient asks probing questions regarding treatment and performance
Anxious	Patients who express/are perceived as anxious, scared, or nervous before, during or after treatment. E.g.: Patient appears anxious, trembles, sweats.
Aggressive	Patients who express anger or aggression through body language or speech. E.g.: Patient raises voice, acts out.
Happy	Patients who express that they are satisfied with the therapist or treatment in the form of body language or speech. E.g.: Patient smiles, expresses gratitude.
Trustful	Patients who express that they trust the therapist and the choice of treatment. Can also be characterized as uncritical. E.g.: Patient appears to rely fully on practitioner, behaves uncritically.
Indifferent	Patients who do not express neither satisfaction nor dissatisfaction, and who may also be disinterested. E.g.: Patient responds poorly, little facial expressiveness.

### Ethics

The study was submitted to the Regional Committee for Medical and Health Research Ethics (REK), which concluded that the project was not health research (reference number 30714/REK nord). All participants were presented with written information about the study as they accessed the electronic questionnaire and had to consent actively to participation before responding to the questionnaire.

### Statistical analysis

The results were analyzed using SPSS version 28 and JASP version 0.16.4.0. Due to the lack of normal distribution for the independent variables the hypotheses were investigated by non-parametric Mann-Whitney U-tests, Kruskal-Wallis H tests, and Spearman rank order correlation.

## Results

Out of the estimated 500 individuals that received the questionnaire, 165 responded, which gave an estimated 33% response rate. Of these 117 were women and 48 men, and there were 39 students and 126 dentists. Participants on average had 11.2 years of experience. See Table 2 for a descriptive summary of the data. The analysis shows no difference between men and women with regards to quality-of-life score (SWLS) or in the perceived frequency of challenging encounters. With regards to dentists versus dental students, the analysis showed no differences between the participating dentists and dental students regarding the extent of perceived challenges in meetings with most patient types. However, a difference was found for aggressive patients, with dental students experiencing these patients as more challenging (*n* = 39; Mdn = 3.00, mean rank = 95.79) than dentists (*n* = 126; Mdn = 2.00, mean rank = 79.04; U = 1958.00, z = −2.12, *p* < 0.05).

**Table 2:** Descriptives; means, standard deviations (SD), medians, and interquartile range (IQR) for study variables.

Variable	Category	*N* (%)
Sex (*n* = 165)	Female	117 (71)
	Male	48 (29)
Occupation (*n* = 165)	Student	39 (24)
	Dentist	126 (76)

Variable	Mean (SD)	Median (IQR)
Age	36.90 (12.43)	34.00 (16.00)
Years of practice (*n* = 163)	11.17 (12.20)	8.00 (13.50)
Satisfaction with Life Scale (*n* = 165)	26.35 (5.78)	27.00 (7.00)
Frequency of challenging encounters (*n* = 165)	2.31 (0.44)	2.33 (0.50)
Total exposure (%) to challenging encounters (*n* = 162)	18.77 (17.10)	15.00 (15.00)

Reflective Functioning	RFQ_C		RFQ_U	
	Mean (SD)	Median (IQR)	Mean (SD)	Median (IQR)
	1.36 (0.72)	1.33 (1.00)	0.33 (0.38)	0.17 (0.50)

The compound mentalization variable constructed from the median scores on the RFQ_C and RFQ_U showed that 38% of participants were “overconfident” with regards to the mental states of other, 37% were “underconfident”, and 14% would be considered having optimal confidence levels (a certain amount of both certainty and uncertainty with regards to the mental states of others). While not included in further analysis due to the theoretical difficulties in interpreting data, 11% of participants would fit within both overconfident and underconfident categories.

**Fig. 1:**
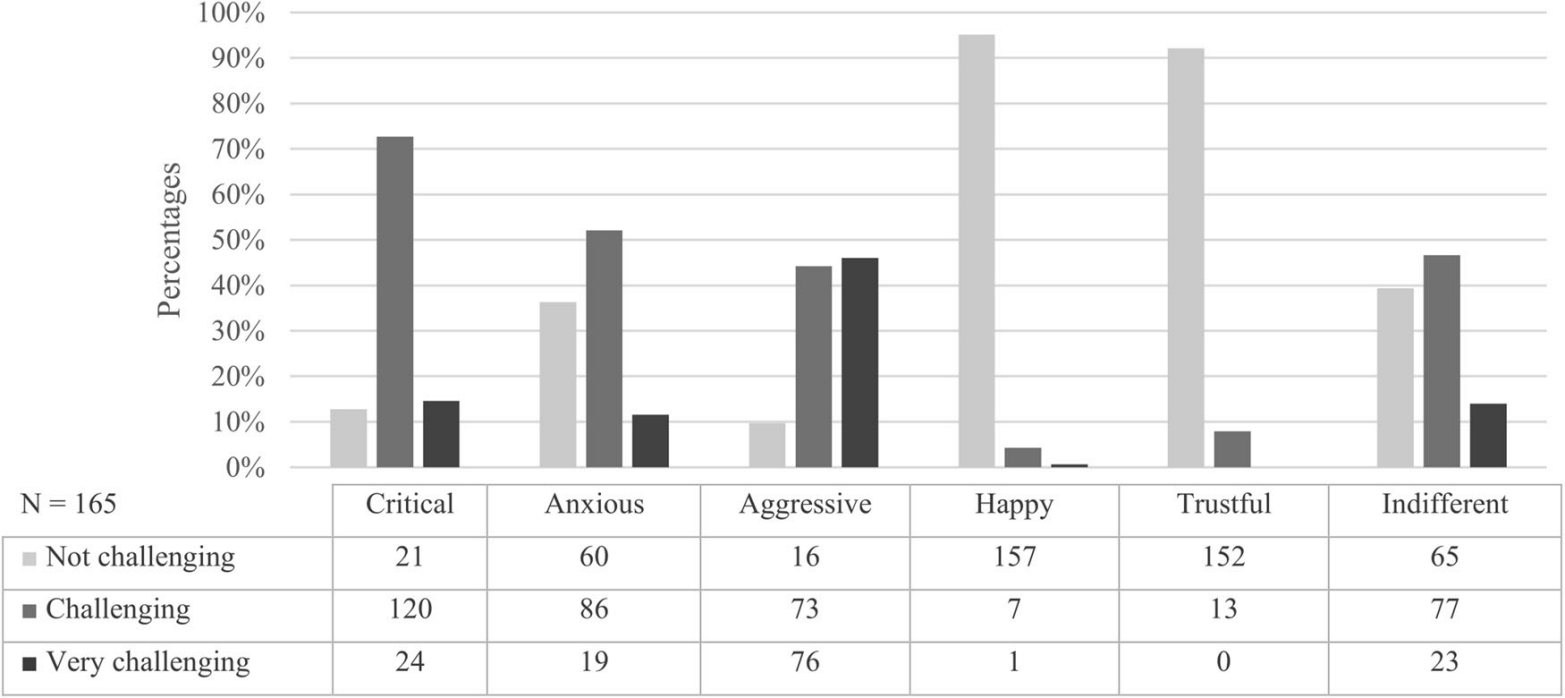
Perception of challenging patient encounters by hypothetical patient traits.

The most challenging encounter reported was with regards to aggressive patients, where 90.3% of the participants perceived these patients as challenging or very challenging. The second most challenging patient trait were critical patients, where 87.2% of the participants reported these as either challenging or very challenging. For anxious patients the majority reported these patients as challenging or very challenging (63.6%), but 36.4% reported them as not challenging. The same goes for indifferent patients (60.7%) where over half of the participants found these patients challenging or very challenging. Only a minority of the participants found trustful patients (7.9%) and happy patients (4.8%) challenging or very challenging (See Fig. 1).

The most common everyday encounter in the clinic was happy and trustful patients, with respectively 81.2% for happy patients, and 80% for trustful patients. It seems like anxious patients is commonly recognized at the clinic, with almost 30% reporting daily encounters, and nearly half of the participants reporting weekly encounters. Most participants (75.2%) reported critical patients as a rare occurrence, with encounters once a month or less often, while aggressive patients were reported as the rarest occurrence at the clinic, with 97% claiming to interact with aggressive patients monthly or less often (See Table 3).

**Table 3:** Frequencies of challenging patient encounters by hypothetical patient traits.

Patient traits	Daily *n* (%)	Weekly *n* (%)	Monthly or less often *n* (%)
**Critical**	8 (4.8)	33 (20.0)	124 (75.2)
**Anxious**	46 (27.9)	77 (46.7)	42 (25.5)
**Aggressive**	0 (0.0)	5 (3.0)	160 (97.0)
**Happy**	134 (81.2)	27 (16.4)	4 (2.4)
**Trustful**	132 (80.0)	28 (17.0)	5 (3.0)
**Indifferent**	24 (14.5)	71 (43.0)	70 (42.4)

### Hypothesis 1: Mentalization and perception of challenging patients

The first hypothesis proposed that mentalization tendencies would influence the perception of patients, with underconfident dental personnel experiencing more challenges related to both hypothetical patient traits and reporting more “total exposure” to challenging patient encounters. The recoded compound variable indicating primary mentalization tendencies (hypermentalization/overconfidence vs genuine mentalization / optimal confidence vs hypomentalization/underconfidence) were used as an independent, group variable in a Kruskal-Wallis H test, with the perception of challenging patient types and the overall percentage estimation of challenging patient encounters as dependent variables (*N* = 147).

The Kruskal-Wallis H test showed that there was a main effect of mentalization tendencies on ratings of perceived challenge related to Critical (H(2) = 9.26, *p* = 0.010) and Anxious patients (H(2) = 7.32, *p* = 0.026), as well as the Percent Estimation of Challenging Patient Encounters (H(2) = 7.01, *p* = 0.030). Mean ranks are provided in Table 4 with higher ranks indicating higher perceived challenge. In order to answer Hypothesis 1, Dunn’s post-hoc comparisons with Bonferroni correction of the mean rank of ratings were made between the three levels of mentalization tendencies to investigate the specific nature of the differences. Interestingly, a similar pattern was identified for all the comparisons made, where overconfident participants rated patient types and total exposure to challenging patients as less challenging than underconfident participants. Concerning the ratings of Critical patients, a significant difference in mean ranks was found between overconfident (mean rank = 64.46) and underconfident (mean rank = 80.88) participants (z = −2.77, *p* = 0.017). Similarly, for ratings of Anxious patients, a significant difference was found between overconfident (mean rank = 64.98) and underconfident (mean rank = 83.63) participants (z = −2.70, *p* < 0.021). Finally, for the Percent Estimation of Challenging Patients a significant difference was found between overconfident (mean rank = 62.30) and underconfident (mean rank = 81.58) participants (z = −2.57, *p* = 0.031). No other differences were identified through the pairwise comparisons. The analysis gave partial support for Hypothesis 1.

**Table 4:** Means, standard deviations, and mean ranks of ratings of how challenging different patient types are perceived, and the estimated percentage total exposure to challenging patients; ordered by mentalization tendencies.

Variables	Hypermentalization			Genuine mentalization			Hypomentalization			*P*
	M	SD	M-rank	M	SD	M-rank	M	SD	M-rank	
Critical	1.86	0.50	64.46	2.13	0.55	81.89	2.10	0.48	80.88	*0.010*
Anxious	1.62	0.68	64.98	1.74	0.62	73.15	1.91	0.63	83.63	*0.026*
Aggressive	2.27	0.65	68.52	2.43	0.59	77.78	2.43	0.65	78.23	0.324
Happy	1.08	0.33	75.19	1.00	0.00	70.50	1.05	0.22	74.09	0.471
Trustful	1.06	0.25	72.67	1.04	0.21	71.20	1.12	0.33	76.43	0.448
Indifferent	1.75	0.76	72.42	1.83	0.72	77.67	1.76	0.66	74.25	0.857
%Exposure	16.01	18.67	62.30	20.00	17.19	77.54	20.27	15.34	81.58	*0.030*
QoL	28.16	5.18	88.17	26.09	5.58	72.11	24.46	5.91	60.08	*0.001*

Tests of main effects were made using Kruskal-Wallis H-Tests. Significant differences in italics.

### Hypothesis 2 Mentalization and quality of life

It was expected that overconfidence with regards to mental states would be associated with higher QoL-scores compared to underconfidence. In order to investigate this hypothesis, a Kruskal-Wallis H test was performed with primary mentalization tendencies (hypermentalization/overconfidence vs genuine mentalization / optimal confidence vs hypomentalization/underconfidence) used as an independent, group variable in a Kruskal-Wallis H test, with the SWLS sum score (QoL) as the dependent variable (*N* = 147). The analysis showed that there was a statistically significant main effect of mentalization tendencies on QoL (H(2) = 13.59, *p* = 0.001; Table 4), and Dunn’s post-hoc comparisons with Bonferroni correction of the mean ranks of QoL were made between the three levels of mentalization tendencies in order to investigate the specific nature of the differences. A significant difference for QoL was found, with overconfident participants reporting higher QoL (mean rank = 88.17) than underconfident participants (mean rank = 60.08; *t* = 3.68, *p* < 0.001). No other significant differences were found.

### Hypothesis 3: Perception of challenging patients and quality of life

The third hypothesis stated that increased exposure to challenging patients would be associated with decreased perceived QoL for dental practitioners. In order to test the hypothesis a one-sided Spearman rank-order correlation was performed to test the association between SWLS scores and the Percent Estimation of Challenging Patient Encounters (“total exposure”). As predicted and in support of Hypothesis 3, the analysis showed a significant negative relationship between the estimated total exposure to challenging encounters and QoL; r(160) = −0.15, *p* = 0.026.

## Discussion

The results show that the mentalization capabilities of dental practitioners are associated with perceptions of both challenging patients and quality of life. Also, a link is identified between challenging encounters and quality of life, where higher scores for quality of life is linked to lower percentages of challenging encounters. Previous studies have supported this association by highlighting the influence of challenging encounters and emotionally demanding patients on stress development and burnout [6, 21, 37], which may be an indicator, or at least an influencing factor, on quality of life. An earlier study reported that 25% of dental patients were perceived as challenging [21], while the results presented in the current study shows that dental practitioners perceive approximately 19% - one in five - of their patients as challenging. Also, the current results provide additional insight about which patient types are perceived by dental practitioners as most challenging. In accordance with former research [21], aggressive patients were found to be the most challenging of the patient types; but also the least often encountered; with nine out of ten participants reporting this patient trait as challenging or very challenging.

In terms of how challenging the study participants perceive critical and anxious patients, there is a difference between participants who hypermentalize and those who hypomentalize, where hypermentalization is associated with lower perceived challenges and hypomentalization higher perceived challenges. In general, people who hypermentalize have a higher-than-normal evaluation of their capacity to identify their own and other people’s mental states and the origins of people’s behavior. This implies that one explanation for the perception of fewer challenges among these participants might be that they have readily available explanatory models for patients’ behavior, and that they are less likely to question the patient’s mental state because they already believe they got the answer or fully understand the situation. This “overconfident attitude” might thus make the situation more comfortable and less prone to cause aversive feelings due to uncertainty, unlike for dental practitioners who hypomentalize. Since this state refers to an individual’s lower-than-normal evaluation of skill and capacity to identify their own and other people’s mental states and behavior, this might lead to an “underconfident attitude” towards patients. In the current study, this state appears to lead to the experience of critical and anxious patients as more difficult to interact with. It might be suggested that hypomentalizing practitioners might feel insecure about how to approach patients, what questions to ask, and might feel unsure on how to react in different situations. The concept of uncertainty has been studied quite extensively in the healthcare setting as a quite common experience irrespective of practice field [38]. Interestingly, when faced with uncertainty in social situations people tend to fall back on automatic and simplified cooperative behaviours, so called social heuristics [17]. This approach are partly aimed at reducing the aversive feelings associated with social unpredictability rather than attempting to solve the uncertainty itself [17, 24]. Thus, it makes sense that dental practitioners who tend to regard social interactions in light of uncertainty might be more interested in reducing the aversive feelings apparent in the situation rather than attempt more effortful modes of social processing (such as perspective taking) [24], which could end up exacerbating the conflict and ultimately worsening the feelings of uncertainty.

Importantly, neither hypermentalization (overconfidence) or hypomentalization (underconfidence) reflect optimal patient handling or accurate assessment of mental states, and both tendencies might lend themselves to criticism and dissatisfaction from patients although possibly for different reasons. Where underconfident and uncertain dentists might be perceived as insecure in their professional role, overconfident and highly certain dentists might be perceived as non-attentive or uninterested in the patient. In contrast, dental practitioners with optimal confidence levels would perhaps be more likely to shift between different modes of social inference in response to the interaction with patients in real time, and perhaps also be more flexible in their response to challenging encounters and how to cope with stressful professional events.

Research has shown that emotional competence to some degree protect against occupational stress [39, 40], correlates positively to subjective well-being and life satisfaction [41, 42], and correlates positively with the ability to handle distress and negative events [43]. Seen in relation to the current results, it is natural to propose that metacognitive skills, such as mentalization and emotional competence, could play important roles in shaping the ability of the dental practitioner to cope with stress and challenging situations, and consequently impact the dentist-patient relationship. For instance, emotional dysregulation has been identified as an important aspect related to occupational burnout among both dental practitioners and dental students [44] and mentalization capabilities appear to increase resilience to stress among healthcare professionals [31]. Also, studies have reported that emotional intelligence among dental students was linked to among other things, life satisfaction [45], which is in line with the results of this study. In line with this, several studies have emphasized the importance of incorporating emotional competence in the curriculum for dental students [45, 46], as well as focusing on continuing professional development packages or initiatives for coping with stress and building personal resilience [47, 48].

Although we believe this is the only study investigating this specific combination of variables, there are analogue findings available. For instance, research on sensory processing sensitivity has shown that dental health professionals’ sensitivity to both internal and external stimuli predict reactions to patients’ stress and trauma, and impact work satisfaction [49]. In the current context it might seem that underconfident dental practitioners similarly appear to be sensitive to vicariously acquiring stress and discomfort from their patients, perhaps due to a lack of overt explanations for the stress and discomfort. While the current study did not include measurements of work satisfaction as such, the total exposure estimation of challenging encounters could be an indirect indication of this. However, mentalization is not a skill limited to the clinical setting, and it could be assumed that the mentalization tendencies present in the current data also relate to the participants’ social functioning more generally, which could explain the effects related to overall life satisfaction.

Finally, while the current results suggest that how dental practitioners approach mentalization might impact both their clinical and private life, the practitioners’ own understanding of the impact and importance of mentalization, as well as how they perceive the phenomenon, is still unknown. It is reasonable, for instance, to assume that dental practitioners that are aware of their own mentalization tendencies, perhaps through inherent interests in behavioral or psychological aspects of dentistry, might be more protected against the pitfalls related to mentalization imbalances in either direction. As suggested by other authors however the insight into what are often implicit processes might vary [30]. Future studies might benefit from investigating specifically how dental practitioners perceive and understand mentalization skills in a clinical setting, which would call for the use of qualitative methods.

### Limitations

The response rate for the study is a rough estimate, since the distribution method did not provide us with information about the exact number of people who received the invitation to participate in the study. Also, the study design made it likely that some selection bias exists by which the study population would mostly include those with a specific interest in the topic under study. Furthermore, care should be taken in interpreting that there are no differences between dental students and dentists with regards to the topics investigated in this study, however the current sample were not able to produce evidence for such differences. Since 11% of participants were categorized as both underconfident and overconfident, it may imply that there is measurement error associated with the RFQ, which has come under criticism for not measuring hypermentalization and hypomentalization to the same degree [50]. Also, recoding RFQ subscales from two ordinal scales to one compound categorical variable would decrease the resolution of the measured concepts, and as a result, increase the chances of mis-categorizations. Finally, care should be taken in interpreting the reported associations between quality of life, mentalization tendencies, and experiences of challenging patient encounters as anything other than associations, as quality of life is a complex and multifaceted concept.

## Conclusions

The encounter between dental practitioners and patients is a social arena with the potential for solving health challenges but also for conflict and emotional distress. The current study indicates an interesting interplay between the mentalization tendencies of dental practitioners, their perceptions of challenging patient encounters, and ultimately how dental practitioners experience quality of life outside of their dental practice. These results highlight the need for increased focus on how dental practitioners handle social and emotional challenges in order to benefit the dental profession and promote positive patient experiences.

## Data Availability

Due to the terms of consent to participation, data will not be made available for other studies.
